# The Effect of Long-Term Azithromycin on Objective and Subjective Cough in Chronic Respiratory Disease: A Systematic Review and Meta-analysis of Randomised Controlled Trials and Noncomparative Studies

**DOI:** 10.1007/s00408-024-00729-8

**Published:** 2024-07-11

**Authors:** Dominic L. Sykes, Pavan Mason, Nithusa Rahunathan, Simon P. Hart, Alyn H. Morice, Michael G. Crooks

**Affiliations:** 1 Respiratory Research Group, Hull York Medical School, Hull, UK; 2 North Middlesex University Hospital NHS Trust, London, UK; 3 North Lincolnshire and Goole NHS Foundation Trust, Scunthorpe, UK; 4 Academic Respiratory Medicine, Castle Hill Hospital, 1st Floor Daisy Building, Cottingham, HU16 5JQ UK

**Keywords:** Azithromycin, Cough, Chronic respiratory disease

## Abstract

**Introduction:**

Azithromycin is an effective treatment for various respiratory conditions but its effect on cough is poorly understood. We synthesised data from randomised controlled trials (RCTs) and noncomparative studies (NCT) examining its effect on objective and subjective cough.

**Methods:**

After prospective registration on PROSPERO, we searched MEDLINE, EMBASE, and CENTRAL for both RCTs and NCT trials examining the effect azithromycin on cough in respiratory disease.

**Results:**

We identified 1240 studies of which 6 (4 RCTs and 2 NCT studies) were included in the meta-analysis, with a total of 275 patients. Azithromycin was associated with significant improvement in Leicester Cough Questionnaire scores at follow-up when compared to baseline scores (SMD = 0.62 [95% CI 0.12 to 1.12], *p* = 0.01). However, when only RCTs were synthesised, no significant effect was observed (SMD = 0.12 [95% CI − 0.36 to 0.60], *p* = 0.62). There was no significant reduction in cough severity VAS score (SMD = − 0.39 [95% CI − 0.92 to 0.14], *p* = 0.15). There was no significant reduction in objective cough count (SMD = − 0.41 [95% CI − 1.04 to 0.32], *p* = 0.09).

**Conclusion:**

Azithromycin therapy improves cough-related quality of life in various chronic respiratory diseases; however, there was no significant effect on cough outcomes when only data from RCTs were synthesised. We believe that to accurately identify which patients whose cough would benefit from azithromycin a large-scale clinical trial of patients with a broad spectrum of respiratory diseases, with sufficiently severe cough, should be undertaken with subgroup analysis of individual disease areas.

**Supplementary Information:**

The online version contains supplementary material available at 10.1007/s00408-024-00729-8.

## Introduction

Chronic respiratory diseases affect one in five people in the United Kingdom and now represent the third leading cause of death in England [1]. Amongst such patients, the symptom of cough is extremely prevalent and often accounts for a significant proportion of symptom burden and is responsible for up to 10% of clinical contacts in primary care [2, 3]. In chronic respiratory diseases such as idiopathic pulmonary fibrosis (IPF), chronic obstructive pulmonary disease (COPD), and asthma, a higher burden of cough as measured by both objective and subjective assessment is associated with disease progression [4–6]. Despite this, the primary outcomes, and indeed secondary outcomes, of large-scale clinical trials are seldom related to objective or subjective measures of cough.

The use of long-term azithromycin has become commonplace in the field of respiratory medicine over the past 10 years. Although its exact mechanism remains debated, it has been shown in various large randomised controlled trials to reduce exacerbation frequency in patients with COPD, asthma, and bronchiectasis [7–9]. In the light of this, the British Thoracic Society (BTS) guideline for the use of long-term macrolides recommends their use in patients who experience frequent exacerbations in each of these diseases [10]. However, this guideline recommends against the use of long-term macrolides in unexplained chronic cough, moreover, the recent BTS clinical statement of chronic cough recommends only using long-term macrolides in those with chronic productive cough and recommends against its use in non-productive cough [11].

The exact mechanism by which macrolides work is incompletely understood; however, the widely accepted theory is of their immunomodulatory properties [12]. The most convincing evidence is of the positive effect of azithromycin on the ability of airway macrophages to phagocytose bacteria, which has been shown in COPD patients [13]. There have been many in vitro studies investigating the effect of macrolide on other aspects of innate and adaptive immunity, with conflicting results [14–17]. A less studied mechanism of azithromycin is that of its effect on gastrointestinal motility, through its potent effect as an agonist of motilin receptors [18]. Previous data have shown that up to two-thirds of patients with chronic respiratory disease and high cough burden have an element of oesophageal dysmotility [19]. This oesophageal dysmotility may well cause non-acidic gaseous refluxate to cause inflammation in upper and lower airways and cause sensitisation of vagal afferents, leading to increased cough reflex sensitivity, known as cough hypersensitivity syndrome [20, 21]. However, the direct impact of azithromycin on objective oesophageal function and its correlation with objective and subjective assessments of cough severity is something that is yet to be studied in sufficient detail.

In this systematic review and meta-analysis of randomised controlled trials (RCTs) and non-randomised noncomparative (NCT) studies, we synthesise the current evidence for the use of azithromycin in chronic respiratory disease. We elected to study azithromycin alone rather than assessing all macrolides as it has the largest clinical trial evidence base for exacerbation reduction in asthma, bronchiectasis, and COPD [10]. We aimed to assess the effects of azithromycin on subjective patient-reported outcomes of cough as well as objective 24-h cough counts.

## Methods

### Protocol Registration

The review protocol was prospectively registered with PROSPERO. With the registration number CRD42023433530.

### Eligibility Criteria

We searched for English language studies of adults (≥ 18 years old) with a diagnosis of chronic respiratory disease including Chronic Obstructive Pulmonary Disease (COPD), Asthma, Chronic Cough, Interstitial Lung Disease (including Idiopathic Pulmonary Fibrosis), and non-cystic fibrosis Bronchiectasis who were being treated with long-term azithromycin. Both randomised controlled trials (RCTs) and non-randomised noncomparative trials (NCT) of efficacy were eligible for inclusion. We excluded trials comparing azithromycin with other long-term macrolide therapy. All common azithromycin treatment regimens including once daily and three times weekly dosing were eligible for inclusion.

### Search Strategy

We searched electronic literature databases MEDLINE, EMBASE, and Cochrane Central Register of Controlled Trials in June 2023 using the terms (1) azithromycin AND, (2) cough AND, (3) asthma; OR, (4) COPD or chronic obstructive pulmonary disease; OR, (5) ILD or interstitial lung disease; OR, (6) IPF or idiopathic pulmonary fibrosis; OR, (7) Chronic Cough or refractory chronic cough or idiopathic chronic cough; OR, (8) Bronchiectasis. Reference lists of previous systematic reviews with similar endpoints were hand-searched for additional titles. The full search strategy, including the results of the searches for each database, can be found in the supplementary materials.

### Study Screening and Selection

All titles, abstracts, and full-text articles were uploaded to the online review website Covidence (www.covidence.org). Title and abstract screening was performed by two reviewers independently (D L Sykes and N Rahunathan) with discrepancies being resolved by a third reviewer (P Mason). Full-text articles of potentially eligible studies were again screened by two independent reviewers (D L Sykes and P Mason) with discrepancies being resolved by consensus. Reasons for exclusion of full-text articles were recorded.

### Outcomes of Interest

To be eligible for inclusion in the analysis, studies had to include at least one of the following as either a primary or secondary outcome. The first main outcome of interest for this review was patient-reported outcome measures of cough including Leicester Cough Questionnaire, Hull Airway Reflux QuestionnaireM, Cough Visual Analogue Scale (VAS), Cough Numerical Rating Scale (NRS), and Cough Quality of Life Questionnaire. The second main outcome of interest for this review was the measure of objective cough counting; studies would be eligible if they used any method of cough counting including (but not limited to) VitaloJAK™, Leicester Cough Monitor, and Hull Automatic Cough Counter.

### Data Extraction

All pre-specified study data were extracted from studies using the Covidence online data extraction tool. Two authors (D L Sykes and N Rahunathan) independently recorded the study data including the following:

- Study Data: first author, year of publication, geographical setting, source of funding
- Methods: study design, study setting, duration of study, inclusion/exclusion criteria, azithromycin dosing regimen, type of cough recorder used, patient-reported outcome measure used
- Results: number of participants, mean age, % male, change in patient-reported outcome measures, change in objective cough count, adverse events, and mortality

Once all data were extracted by each author, discrepancies were resolved by consensus and the limitations of each study were discussed.

### Risk-of-Bias Assessment

All studies were assessed for risk of bias by two independent reviewers (D L Sykes and N Rahunathan) using the Cochrane Risk-of-Bias Tool on the Covidence online review website [22]. This tool assesses each study for sequence generation, allocation concealment, blinding of participants, personnel and outcome assessors, incomplete outcome data, selective outcome reporting, and other sources of bias. Studies were also rated on their method of outcome measurement, analysis of groups, and statistical analysis.

NCT studies were assessed for risk of bias using the ROBINS-I risk-of-bias tool. Disagreements in assessment were resolved by consensus.

### Statistical Analysis

All continuous data from patient-reported outcomes and objective cough counts were inputted as raw data in all meta-analyses in this review. The reported outcome of all meta-analyses was standardised mean difference (SMD). Meta-analysis was performed for all studies comparing the baseline mean (SD) and post-treatment follow-up mean (SD) of each outcome in the azithromycin treatment group.

Separate meta-analyses were performed for RCT data where the mean difference (SD) of each outcome between placebo and azithromycin groups was available. Where standard deviations were not available for a particular outcome, they were imputed using a correlation coefficient from a different study in the review, as per the Cochrane Handbook [23].

As there were only a small number of studies included in the final review, we decided not to use *I*^2^ as a measure of heterogeneity as it is biased in small meta-analyses. Due to the differences in diseases examined in the included studies and methods of assessment between the studies included, all meta-analyses were conducted using a random-effects model. All data analyses were performed using IBM SPSS Statistics 28 (IBM Corp., Armonk, NY, USA).

## Results

### Systematic Review

#### Study Selection

**Fig. 1:**
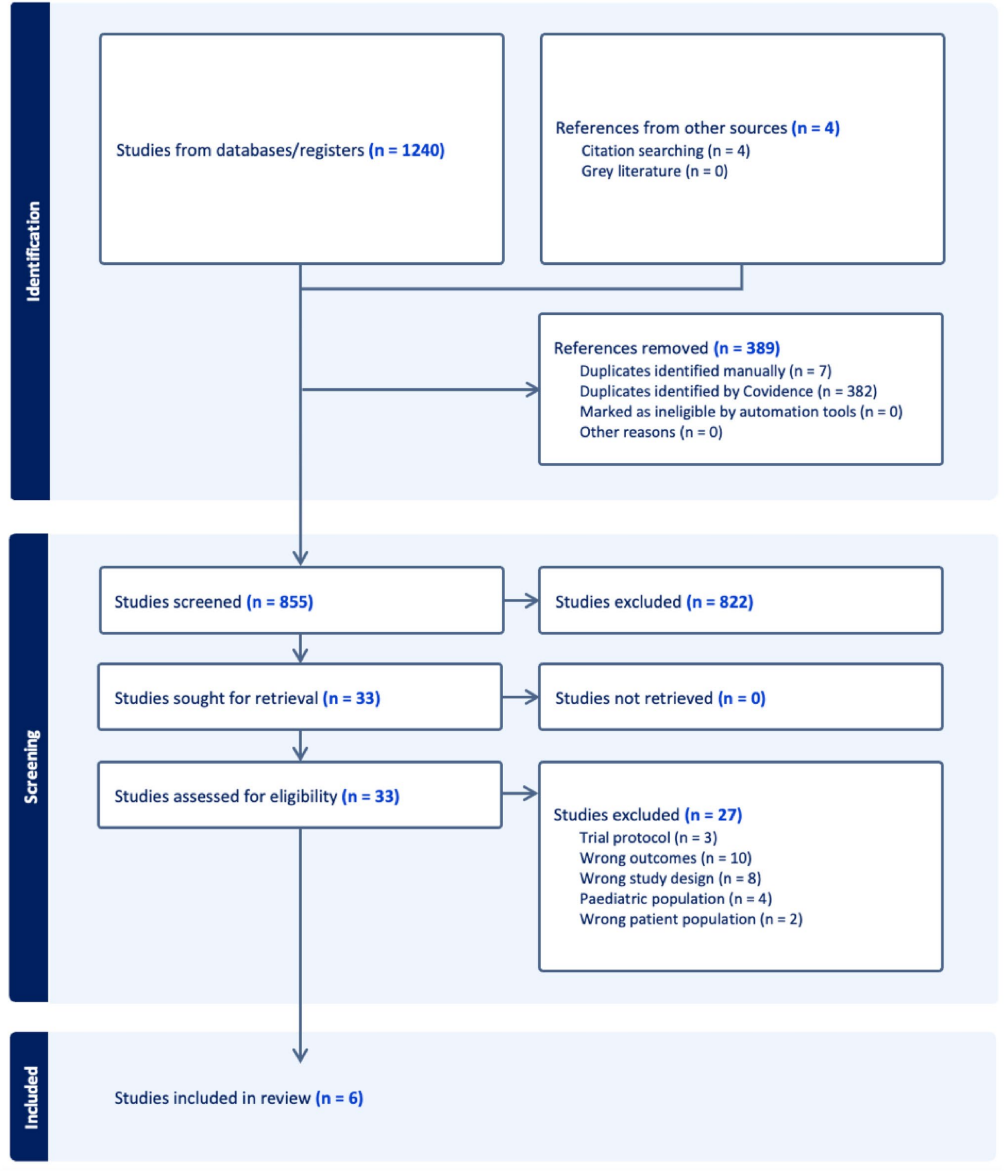
PRISMA flow diagram showing quantities of studies excluded at each stage in the review

The systematic search identified 1240 eligible studies, of these 389 were duplicates. A further 4 studies were included from citation searching, resulting in 855 studies eligible for screening by their title and/or abstract. From these titles, a full-text review was carried out on 33 studies, of which 6 studies met the criteria for inclusion in the final analyses. The Preferred Reporting Items for Systematic Reviews and Meta-Analyses (PRISMA) flow diagram can be visualised in Fig. 1.

#### Study Characteristics

**Fig. 2:**
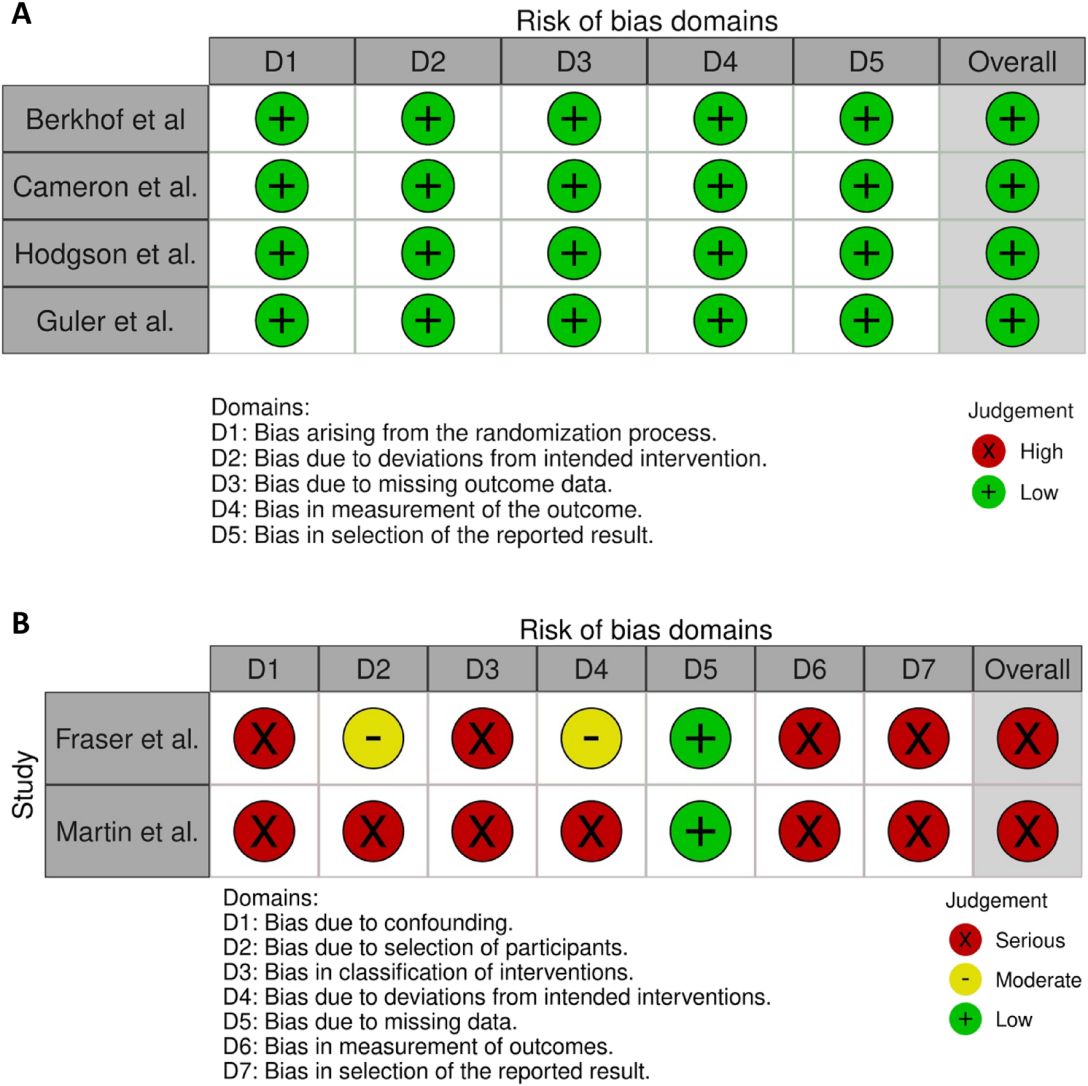
**A** Risk-of-bias assessment for all RCTs included in this review. **B** Risk-of-bias assessment for all NCTs included in this review

A total of 6 studies (*n* = 275) were included in the final analysis, including 4 RCTs [24–27] (*n* = 224) and 2 NCTs [28, 29] (*n* = 51); all 4 RCTs were compared long-term azithromycin with placebo. Table 1 illustrates the characteristics of all included studies. Amongst the RCTs, the risk of bias was rated as low in all categories. As the same risk-of-bias checklist was applied to all studies, the risk of bias was significantly higher in the NCT trials as they were both open-label and did not have a comparator group. Figure 2 shows the risk-of-bias assessment for all studies.

**Table 1:** Characteristics of studies included in the meta-analysis

Study	Condition	Study design	Azithromycin Regimen	Outcomes of interest	Follow-up	Mean Baseline LCQ Score	Number of participants
Fraser et al. (2020)	Sarcoidosis *with* *associated* *cough*	NCT pre–post-clinical trial	250 mg once daily	LCQ, cough severity VAS, 24 h cough counting	*12* *weeks*	15.96	21
Martin et al. (2019)	Chronic Cough *of* *various* *aetiologies,* *including* *asthma,* *GORD,* *and* *early* *bronchiectasis*	NCT pre–post-clinical trial	250 mg three times per week	LCQ	12 weeks	11.5	30
Guler et al. (2021)	Idiopathic pulmonary fibrosis	Randomised placebo-controlled crossover trial	500 mg three times per week	LCQ, cough severity VAS, SGRQ, 24-h cough counting	12 weeks on both placebo and azithromycin	11.6	25
Hodgson et al. (2016)	Chronic cough	Randomised placebo-controlled trial	Azithromycin 500 mg daily for 3 days followed by 250 mg 3 times a week	LCQ, cough severity VAS	12 weeks	10.85	44
Cameron et al. (2013)	Asthma	Randomised placebo-controlled trial	250 mg once daily	LCQ	12 weeks	16.61	71
Berkhof et al. (2013)	COPD	Randomised placebo-controlled trial	250 mg three times per week	LCQ, SGRQ	12–18 weeks	13.95	84

The two NCT studies that were included in the review were both scored as having a ‘serious’ risk of bias using the ROBINS-I tool. The exact scoring for both Fraser et al. and Martin et al. can be seen in the Supplementary Materials.

Of the 4 RCTs included in the analysis, one trial examined the use of azithromycin in COPD, asthma, chronic cough, and IPF. Of the 2 NCT trials, one trial was conducted in sarcoidosis and one in chronic cough.

#### Randomised Controlled Trials

*Berkhof* et al*.*'s study was the largest study identified in the systematic review, examining the role of azithromycin in 84 COPD patients. It demonstrated a significantly greater mean increase in LCQ total score after 12 weeks in the azithromycin group compared with placebo (mean difference 1.3 [95% CI 0.3–2.3] *p* = 0.01), meeting the minimally clinical important difference for the LCQ. There was also a significant improvement in SGRQ total score over 12 weeks with azithromycin compared with placebo mean difference -7.4 [95% CI − 12.5; − 2.5], *p* = 0.004).

Cameron et al.'s study was conducted in a group of 71 smokers with asthma. No effect was seen on mean LCQ following 12 weeks of treatment with azithromycin. The mean difference was -1.06 [95% CI − 2.16 to 0.05], *p* = 0.06.

Hodgson et al.'s study was a trial of 44 patients with chronic cough. There was statistically significant improvement in LCQ score in the azithromycin group from 10.2 to 12.6 (mean change 2.4; 95% CI 0.5 to 4.2; *p* = 0.01), which was not seen in the placebo group (mean change 0.7; 95% CI − 0.6 to 1.9). However, the between-group difference was only observed at 4 weeks and not past this point (mean difference, 1.9 [95% CI 0.1 to 3.8] *p* = 0.04). There was no significant difference between azithromycin and placebo in cough severity VAS scores (*p* = 0.21).

Guler et al. observed the effect of azithromycin in 25 patients with IPF. This study found no difference in total LCQ score (mean difference 0.68 [95% CI − 0.64 to 1.99], *p* = 0.29). There was no difference between cough severity VAS scores (mean difference 0.25 [95% CI − 1.12 to 1.63], *p* = 0.70). 24-h cough recording demonstrated no difference in coughs per hour between placebo and azithromycin (mean difference − 3.9 [95% CI *−* 10.2 to 2.3], *p* = 0.19).

#### Noncomparative Trials

Martin et al. included 30 patients with chronic cough. This showed a significant improvement in LCQ at 12 weeks with a median improvement of 6.3 (*p* < 0.001).

*Fraser* et al*.* *recruited* *21* *patients* *with* *sarcoidosis* *and* *troublesome* *cough.* *This* *study* *showed* *a* *significant* *improvement* *in* *LCQ* *at* *3* *months* *(median* *change,* *1.85* *[−* *1.17* to *12.18],* *p* = *0.006).* There was also a significant improvement in Cough VAS (median change, *−* 9.0 [*−* 93 to 20], p = 0.009). A comparison of 24-h cough counts showed a significant reduction from 228 (43–1950) at baseline to 81 (16–414) at 3 months (*p* = 0.002).

### Meta-analyses

#### Leicester Cough Questionnaire

**Fig. 3:**
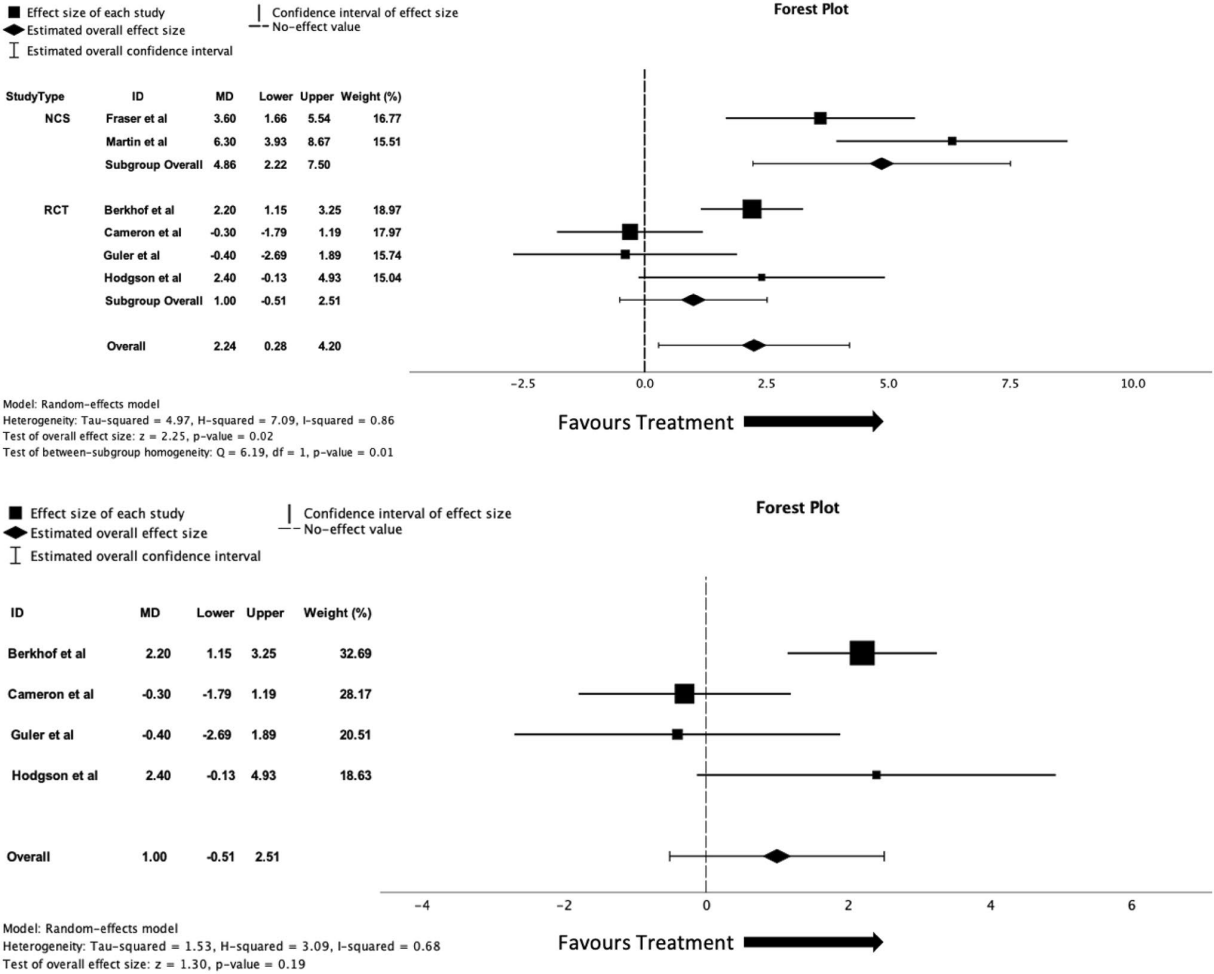
**A** Forest plot of all study data comparing baseline and follow-up LCQ scores with treatment of azithromycin. **B** Forest plot of randomised controlled trials data comparing LCQ score means changes between both azithromycin with placebo groups

All 6 studies included in the analysis recorded LCQ scores at baseline and post-treatment follow-up. The meta-analysis found a significant improvement in LCQ scores with azithromycin treatment when compared to baseline scores (MD = 2.24 [95% CI 0.28–4.20], *p* = 0.02, *I*^2^ = 0.86). When the RCTs were analysed alone, with a comparison of azithromycin vs placebo, there was no significant improvement of LCQ scores (MD = 1.0 [95% CI − 0.51 to 2.51], *p* = 0.19, *I*^2^ = 0.68). Forest plots for the meta-analyses of all studies and for RCTs alone can be seen in Fig. 3.

#### Cough Severity Visual Analogue Scale

**Fig. 4:**
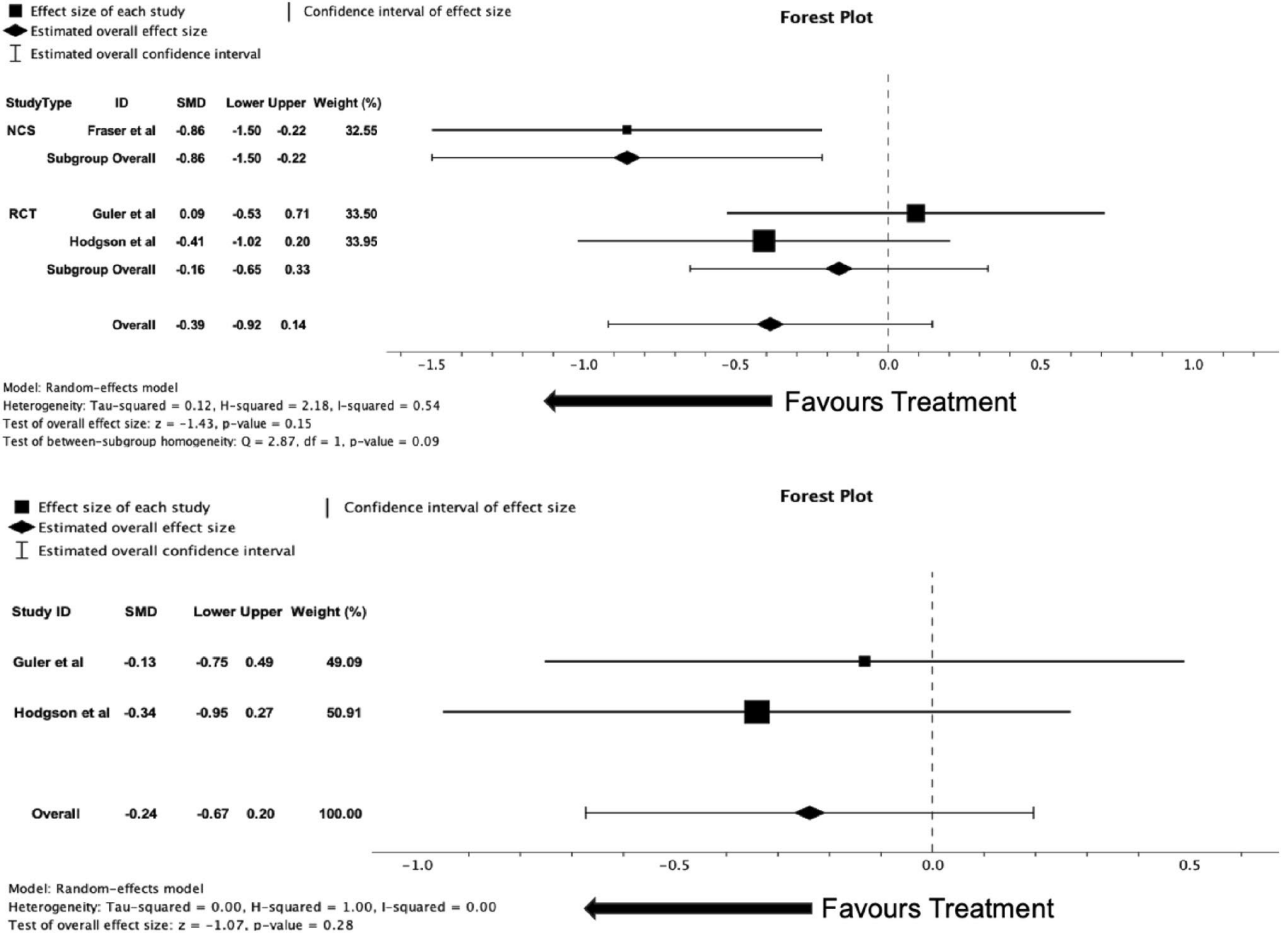
**A** Forest plot of all study data comparing baseline and follow-up cough severity VAS scores with treatment of azithromycin. **B** Forest plot of randomised controlled trials data comparing cough severity VAS score means changes between both azithromycin with placebo groups

Three studies (*n* = 85) included in the analysis measured cough severity VAS as an outcome, 2 RCTs (*n* = 64) and 1 (*n* = 21) NCT study. When all studies were analysed together, there was no effect for reduction of cough severity VAS score with azithromycin treatment compared with baseline score (SMD = -0.39 [95% CI − 0.92 to 0.14], *p* = 0.15, *I*^2^ = 0.54). When RCTs were analysed alone, comparing the effect on cough severity VAS mean difference in azithromycin and placebo groups, there was no significant difference between groups (SMD = − 0.24 [95% CI − 0.67 to 0.20], *p* = 0.28, *I*^2^ = 0.00). It is important to note that the cough severity VAS that was used in *Guler* et al*.* was actually a numerical rating scale (NRS); however, due to the similarity in the nature of assessment, it has been included in the meta-analysis. Forest plots for the meta-analyses of all studies and for RCTs alone can be seen in Fig. 4.

#### St George’s Respiratory Questionnaire

**Fig. 5:**
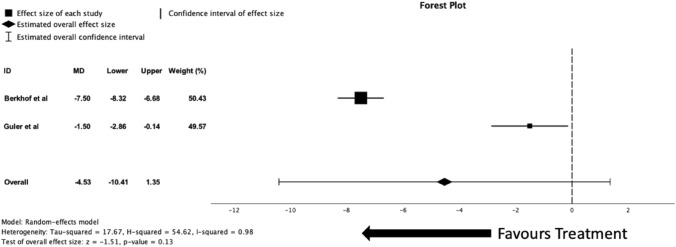
Forest plot of randomised controlled trials data comparing SGRQ score means changes between both azithromycin with placebo groups

Two studies (*n* = 104) included in the analysis measured the SGRQ as an outcome, both were RCTs. There was no significant reduction in SGRQ scores after a meta-analysis of the two studies (MD = -4.53 [95% − 10.41–1.35], *p* = 0.13, *I*^2^ = 0.98). The forest plot for the meta-analysis of these two studies can be seen in Fig. 5.

#### Objective Cough Counts

**Fig. 6:**
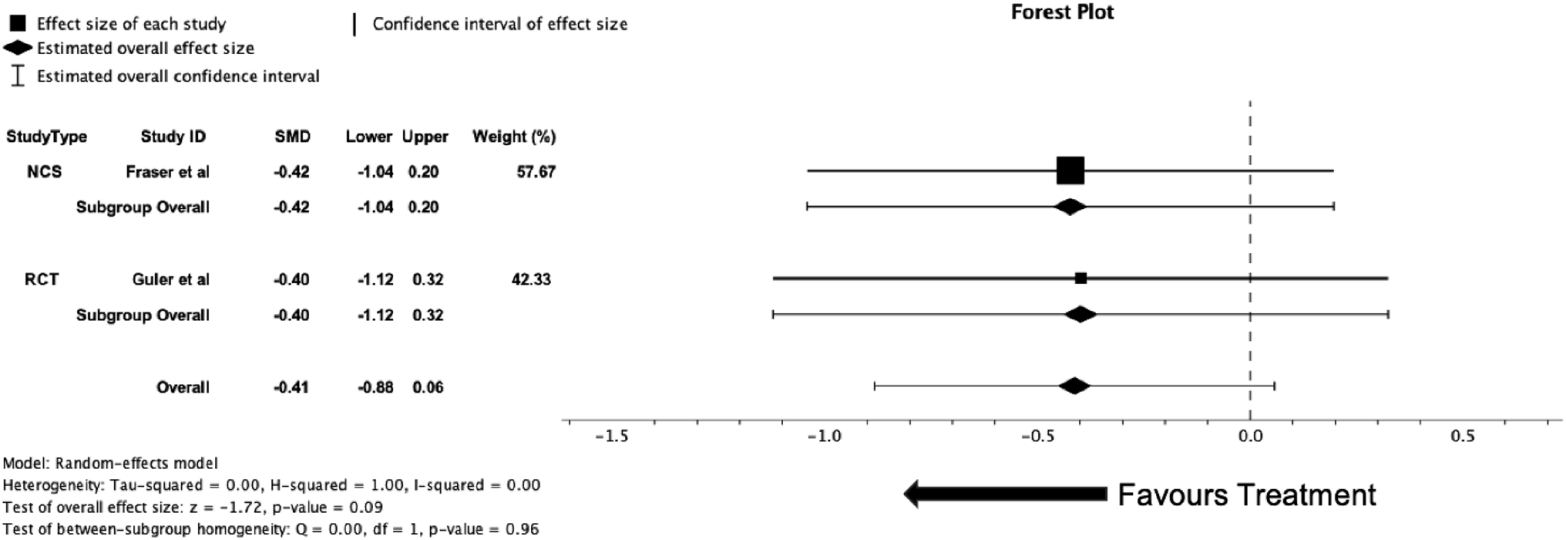
Forest plot of studies with data comparing mean difference of coughs per hour on 24-h cough monitoring at baseline and follow-up with azithromycin treatment

Two studies (*n* = 41) included in the analysis performed 24-h cough recording at both baseline and follow-up after treatment with azithromycin. Fraser et al*.* utilised the Hull Automatic Cough Counter with the Leicester software algorithm for identifying coughs. Guler et al. recorded coughs with the NOX T3 device with Noxturnal software. There were differences in reporting the objective cough counts, with Fraser et al*.* reporting 24-h cough counts and *Guler* et al*.* reporting cough index (i.e. coughs per hour); for the meta-analysis, data were converted into cough index. There was no reduction in cough index in the meta-analysis (SMD = − 0.41 [95% CI − 1.04 to 0.32], *p* = 0.09, *I*^2^ = 0.00). The forest plot for these data can be seen in Fig. 6.

## Discussion

In this systematic review, we have pooled data from 4 RCTs and 2 NCT studies comprising 275 patients with various chronic respiratory diseases. We have shown a significant improvement in cough-related quality of life as measured by the LCQ when comparing pre-treatment baseline values with post-azithromycin treatment follow-up assessments (SMD = 0.62 [95% CI 0.12–1.12], *p* = 0.01). However, when placebo-controlled data from the 4 RCTs were pooled together, there was no statistically significant improvement with treatment over placebo.

The absence of improvement in LCQ scores and objective cough counts in the RCT-only meta-analysis compared to the improvements observed in the NCT studies are in keeping with the large placebo responses that have been observed in previous trials in chronic cough patients [30]. However, this response has not been seen in recent trials examining cough in IPF patients [31, 32]. When analysing the LCQ results from studies included in this review individually, there are 2 RCTs with improvement, 2 RCTs with no difference, and 2 NCT trials showing improvements. The heterogeneity in the findings observed may be attributable to patient selection, for example, Cameron et al*.* included asthmatic patients with a baseline LCQ of 16.6 in the treatment group and 16.9 in the placebo group. These values almost reach the cut-off for normality, which has previously been reported as 17.68 [33]. Furthermore, the two RCTs that showed significant improvement in LCQ scores had patients with baseline LCQ scores of 10.2 and 14.5 (treatment groups), which would indicate that patients with more severe cough would have a higher chance of responding to azithromycin. We have also observed variation in response based on the disease studied in each of the studies, as the positive RCTs were in groups of chronic cough and COPD patients, whereas the negative RCTs were in patients with asthma and IPF, and the two positive NCT studies were in patients with chronic cough and sarcoidosis. We believe that this review ultimately highlights the lack of certainty around the efficacy of azithromycin for the treatment of cough in chronic respiratory disease. It is not clear from the studies included in this review which cohort of patients are set to benefit from long-term treatment.

Of the studies included in our final analysis, only two trials with a combined total of 41 patients utilised the technique of objective cough counting as one of the outcomes of their study. Both studies showed a reduction in cough counts with the use of azithromycin, and although our analysis did not show this to be statistically significant, we believe that this is due to a lack of statistical power from the small sample sizes in the individual studies and therefore in the meta-analysis. Both studies investigated azithromycin in interstitial lung disease with Sarcoidosis in Fraser et al. and IPF in Guler et al*.* The data from both included studies represent a promising signal which warrants further investigation of the effect of long-term macrolides on objective cough in different respiratory diseases. Objective assessment of cough through 24-h cough counting has been shown to correlate with more traditional outcome measures of disease control respiratory disease and has been shown to predict improvement in disease control in asthma [34], COPD [35], and IPF [36]. Traditionally, clinic trials of novel pharmacological interventions for idiopathic/refractory chronic cough have used this metric as their primary outcome [30] and it has more recently been utilised as a primary outcome for trials of new therapeutics in IPF [31]. Despite its increasing usage, the optimal method of objective cough counting remains the centre of debate [37, 38]. Indeed, the two trials included in this review reported different metrics, with Guler et al. reporting cough index (coughs per hour) and Fraser et al*.* reporting 24-h cough count. Other reported cough metrics include awake cough frequency, which is more applicable in idiopathic/refractory chronic cough as such patients tend not to cough as much overnight but is less applicable to asthmatic patients who may well cough mostly throughout the night. More novel techniques of cough assessment are being explored, such as ambulatory and home continuous cough monitoring [39, 40].

There are several limitations in this systematic review and meta-analysis. Firstly, there is a relative dearth of available studies that examine the impact of azithromycin on cough; furthermore, the included studies boast only small-moderate sample sizes. This review aimed to widen the number of participants by including NCT studies as well as RCTs which will of course alter the reliability of the results as such studies are not controlled or blinded which will introduce several biases that are not present in reviews of RCTs only. For this reason, we performed additional analysis on RCTs separately to gain further understanding of the effect of azithromycin compared with placebo on subjective measures of cough. Another potential limitation of this analysis is the heterogeneity of the patient population from which these studies were selected, as different respiratory diseases have distinct pathophysiology and differential cough burden, which may affect the efficacy of azithromycin. Moreover, there was heterogeneity in certain aspects of the study protocols, such the method of azithromycin dosing and objective cough measurement techniques. Despite these limitations, this systematic review was prospectively designed, registered, conducted, and reported in line with PRISMA guidelines.

In conclusion, we have demonstrated that long-term azithromycin therapy improves cough-related quality of life in various chronic respiratory diseases; however, the data from randomised controlled trials did not support this finding. We believe that to accurately identify which patients whose cough would benefit from azithromycin a large-scale clinical trial of patients with a broad spectrum of respiratory diseases, with sufficiently severe cough, should be undertaken with subgroup analysis of individual disease areas.

## Supplementary Information

Below is the link to the electronic supplementary material.

Supplementary file1 (DOCX 494 KB)
